# Impacts of *M. oleifera* Leaf Extract on Weight Loss, the Metabolomic Profile, and the Intestinal Microbiota in Individuals With Simple Obesity

**DOI:** 10.1002/fsn3.72292

**Published:** 2026-08-28

**Authors:** Xiaoxian Zhou, Daoqiang He, Jia Xiong, Xinle Zhang, Yongfang Zhou, Jia Luo, Lan Zhou, Huilin Chen, Jingwen Dao, Fei Mi, Yanjiao Wang, Shaoxiong Wu, Hongmei Pan

**Affiliations:** ^1^ Yunnan Provincial Key Laboratory of Public Health and Biosafety and School of Public Health Kunming Medical University Kunming Yunnan China; ^2^ Laboratory Department Fuquan City Center for Disease Control and Prevention (Fuquan City Health Supervision Station) Fuquan Qiannan Buyei and Miao Autonomous Prefecture, Guizhou China; ^3^ Clinical Nutrition Department of Yunnan Cancer Hospital, The Third Affiliated Hospital of Kunming Medical University Peking University Cancer Hospital Yunnan Kunming Yunnan China; ^4^ College of Medicine and Life Sciences Zhangjiajie College Zhangjiajie Hunan China

**Keywords:** gut microbiota, metabolomic profiles, *Moringa oleifera* leaf extracts, simple obesity, weight loss

## Abstract

*Moringa oleifera* leaves, recognized as a novel food resource in China, have shown potential health benefits. To investigate the effects of *Moringa oleifera* leaf extract on weight loss and its underlying mechanisms, this study conducted a randomized, double‐blind clinical trial. In this trial, 35 participants were participated to the placebo group (Con group) or the *M. oleifera* leaf extract group (Med group) for 90 days. Both groups received dietary/lifestyle guidance. Body composition was assessed monthly, and stool and serum samples were collected for 16S ribosomal RNA sequencing (16S rRNA sequencing), biochemical analysis, and metabolomics. The results show that significant changes were observed in body mass index (BMI), basal metabolic rate, muscle mass, and fat‐free mass between the two groups (all *p* < 0.05). Compared with the Con group, the Med group showed a more pronounced decrease in BMI, yet better preserved its basal metabolic rate, muscle mass, and fat‐free mass. Among the metabolic markers, the Med group showed a significant decrease in glycated hemoglobin (HbA1c) and a significant increase in high‐density lipoprotein cholesterol (HDL‐C) compared with the Con group (all *p* < 0.05). Gut microbiota analysis showed there were significant differences in the abundance of 9 taxa between the two groups (*p* < 0.05). Differentially expressed metabolic pathways are concentrated in “Metabolic pathways,” Acetate levels in the feces of the Med group were significantly lower than those in the Con group; untargeted metabolomics identified a total of 132 differentially expressed metabolites, which were enriched in multiple lipid metabolic pathways. There is a certain correlation between the levels of certain differential metabolites and gut microbiota and the expression of clinical indicators. In summary, *M. oleifera* leaf extract can effectively reduce the BMI in obese patients while better preserving basal metabolic rate, muscle mass, and fat‐free mass. HbA1c and HDL‐C levels in the body also showed significant improvement, and the extract played a regulatory role in the abundance of gut microbiota and metabolites related to lipid metabolism.

**Trial Registration:** Chinese Clinical Trial Registry CTR2400089558

## Graphical abstract

A total of 63 subjects participated in the formal trial, randomly assigned to either the control group (*n* = 29) or the intervention group (*n* = 34). The trial period lasted 90 days, during which the intervention group consumed *Moringa oleifera* leaf extract while the control group received a corresponding placebo. Body composition measurements were monitored monthly throughout the study. Ultimately, 17 and 18 subjects from the intervention and control groups, respectively, completed the entire trial protocol. Blood samples were collected from these subjects for analysis of glucose and lipid metabolism‐related indicators, non‐targeted metabolomics, and gut microbiota, with intergroup comparisons conducted.

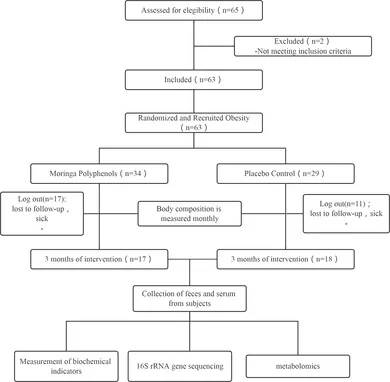

**Abbreviations**
16SrRNA: 16S ribosomal ribonucleic acid
BMI: body mass index
BMR: Basal metabolic rate
Con group: placebo group
DNA: deoxyribonucleic acid
dNTP: deoxyribonucleoside triphosphate
FFM: Fat‐free weight
HbA1c: glycated hemoglobin
HDL‐C: high‐density lipoprotein
Med group:
*Moringa oleifera* leaf extracts group
PCR: polymerase chain reaction
SCFAs: short‐chain fatty acids
SLM: Muscle mass
TG: triglyceride

## 1 Introduction

Obesity/overweight, characterized by the World Health Organization (WHO) as the abnormal accumulation of fat in the body, poses a significant threat to human health. Since 1980, the global prevalence of obesity/overweight has nearly doubled, and one out of every three people is now considered overweight. This trend is increasingly affecting younger people, making obesity a major public health concern and a life‐threatening condition (Zhu et al. 2022; Lin and Li 2021; Jebeile et al. 2022). Obesity can increase the risk of numerous noncommunicable diseases in humans, such as hypertension, dyslipidaemia, type 2 diabetes mellitus (T2DM), cardiovascular disease, osteoporosis, asthma, and various malignancies, and can impact nearly all organs and systems (Safaei et al. 2021; De Lorenzo et al. 2019). Several factors contribute to the development of obesity, including genetics, lifestyle habits, dietary habits, feeding practices, and the gut microbiota (Endalifer and Diress 2020). At present, measures for preventing and controlling obesity include dietary interventions, pharmaceutical interventions, lifestyle changes, and surgical approaches. Conventional weight loss drugs include liraglutide, somatostatin, and orlistat, among others (Chao et al. 2021; Aaseth et al. 2021) However, pharmaceutical interventions and surgery may have adverse effects; for example, orlistat can cause diarrhea. Therefore, there has been increasing interest in the use of natural, active food components in weight loss research.

Gut microbiota are closely associated with the development of several diseases, including neurodegenerative diseases, schizophrenia, and obesity (Cryan et al. 2019). The ratio of the abundance of thick‐walled bacteria to that of *Bacteroidetes* has been used as a marker of susceptibility to obesity: the higher this ratio is, the greater the risk of fat accumulation. Compared with nonobese individuals, individuals with obesity exhibit higher relative abundances of *Prevotellaceae* and *Enterobacteriaceae*; lower relative abundances of *Christensenellaceae*, *Methanobacteriales*, *Lactobacillus*, Bifidobacteria, *Akkermansia*, and other beneficial flora; and reduced diversity in their gut microbiota (Tseng and Wu 2019). Gut microbiota regulate the absorption of energy and the storage of fat (Abenavoli et al. 2019) and affect the secretion of appetite‐related hormones such as GLP‐1, ghrelin, PYY, and LEP (Asadi et al. 2022; Liu et al. 2021). Given these links between the gut microbiota and obesity, the use of probiotics has become a hot topic in the control of obesity.

*Moringa oleifera* leaf extracts are widely used in various products, such as foods, cosmetics, pharmaceuticals, and livestock feed, and exhibit anti‐inflammatory, antioxidant, antibacterial, hypotensive, hepatoprotective, antiulcer, diuretic, antidiabetic, and anticancer effects (Rode et al. 2022; Hodas et al. 2021). Studies have shown that *M. oleifera* leaf extracts can significantly alleviate metabolic disorders in obese rodents fed a high‐fat diet by lowering resistin levels, increasing lipocalin levels and mitigating increases in body weight and visceral fat accumulation through the regulation of lipid metabolism, leptin, and obesity‐related genes (Wu et al. 2021; Alkhudhayri et al. 2021; Kilany et al. 2020; Kundimi et al. 2020). In addition, *M. oleifera* leaf extract can increase the content of beneficial bacteria while decreasing the abundances of harmful bacteria in the feces of obese animal models and improve body weight, lipid levels, and insulin resistance. These findings suggest that *M. oleifera* leaf extract may improve lipid metabolism by regulating the gut microbiota (Li et al. 2022).

Although *M. oleifera* leaf extracts have been shown to induce weight loss, alleviate metabolic disorders, and increase the abundance of beneficial gut microbiota in in vitro and in vivo experiments, there is insufficient evidence for these roles in humans. Therefore, the goal of the present study was to investigate the effects of *M. oleifera* leaf extract on weight loss, metabolism, and the gut microbiota in a sample of individuals with simple obesity.

## 2 Materials and Methods

### 2.1 Study Design

This study was a randomized, double‐blind, placebo‐controlled trial involving volunteers with simple obesity recruited from Yunnan Cancer Hospital (The Third Affiliated Hospital of Kunming Medical University) who were randomly included to the *M. oleifera* extract group (Med group) or the placebo group (Con group).

### 2.2 Subjects

Following application of the inclusion and exclusion criteria below, a total of 63 volunteers with obesity were finally included; they were subsequently randomly divided into the two groups: Con group (*n* = 29) and Med group (*n* = 34).

The inclusion criteria were as follows: (1) body mass index (BMI) ≥ 28 kg/m^2^; (2) no other chronic illnesses; and (3) an understanding of the study's procedures and readiness to engage in the research.

The exclusion criteria were as follows: (1) refusal to provide informed consent; (2) the presence of a chronic disease other than obesity; and (3) involvement in another clinical study within the past 30 days.

#### 2.2.1 Diagnostic Criterion

BMI, recognized worldwide as a metric for assessing obesity, is typically computed as weight (in kilograms) divided by the square of height (in metres). The participants in this study had simple obesity exhibited BMI values meeting the diagnostic criteria for obesity according to the Chinese BMI reference criterion independent of any endocrine disorders (Table 1).

**TABLE 1:** BMI classification in China.

Classification	BMI (kg/m^2^)
Obesity	≥ 28
Overweight	24–27.9
Normal weight	18.5–23.9
Underweight	Under 18.5

### 2.3 Interventions

*M. oleifera* leaf extract was obtained from Yunnan Agricultural University. The Con and Med groups were administered placebo packs and *M. oleifera* leaf extract packs, respectively; the specific compositions of these packs are outlined in Table 2. On the basis of previous research and the outcomes of animal trials, the recommended dosage of *M. oleifera* leaf, a novel food resource, is 4 g per 60 kg of body weight per day. In our trial, each pack of placebo/ *M. oleifera* leaf extract was dissolved in 100–200 mL of lukewarm water. The participants consumed two packs a day, one before breakfast and one before dinner. The entire intervention period spanned 3 months, during which dietitians advised the participants on dietary habits and lifestyles.

**TABLE 2:** Pack ingredients.

Pack name	Net content (g/pack)	Ingredients
Placebo	8	50% peach powder +50% isomaltose
*Moringa oleifera* leaf extract	8	25% *Moringa oleifera* leaf extract +25% peach powder +50% isomaltose

### 2.4 Outcome Indicators

In this study, for each participant, we assessed body composition and various biochemical parameters, including lipid and blood glucose levels and liver and kidney function indicators (for details on the laboratory methods used for the clinical indicators in this study, see Table S1). Fasting blood and stool samples were also collected for 16S ribosomal RNA sequencing (16S rRNA sequencing) and metabolomics analyses.

### 2.5 16S rRNA Gene Sequencing

Fecal DNA was extracted using FastPure Fecal DNA Extraction Kits (MJYH, Shanghai, China). The V3–V4 regions of the 16S rRNA gene were subject to PCR amplification using primer pairs containing barcode sequences. Library preparation of the amplified products was carried out using a NEXTFLEX Rapid DNA‐Seq Kit, and sequencing was performed on an Illumina NextSeq 2000 platform. Quality control of the raw paired‐end sequencing reads was performed using fastp software (version 0.19.6); assembly was conducted using FLASH software (version 1.2.11), and the quality‐controlled and assembled sequences were clustered into operational taxonomic units (OTUs) on the basis of 97% similarity and subject to chimaeras removal with UPARSE v7.1 software. Intergroup differences in alpha diversity were analyzed using Welch's *t*‐test, followed by FDR‐based multiple comparison correction; *p* < 0.05 was considered to indicate statistical significance (ace, chao, sobs, coverage, Shannon, and Simpson). Bray–Curtis distance‐based PCoA (principal coordinate analysis) was used to assess the similarity of microbial community structures across samples, and PERMANOVA nonparametric tests were used to determine whether differences in microbial community structures between sample groups were statistically significant. Using the Wilcoxon rank‐sum test with FDR‐based multiple comparison correction, the between‐group differences were statistically analyzed. Furthermore, we employed linear discriminant analysis‐effect size (LEfSe) (LDA > 2, *p* < 0.05) to identify bacterial taxa exhibiting significant abundance differences from the phylum to the genus level across different groups.

### 2.6 Untargeted Metabolomics Analysis

The Thermo Fisher Scientific's UHPLC‐Exploris 240 system, which combines ultrahigh‐performance liquid chromatography with tandem Fourier transform mass spectrometry, was used to perform Liquid chromatography‐mass spectrometry (LC–MS). Raw data were imported into the metabolomics processing software Progenesis QI v3.0 (Waters Corporation, Milford, USA) for baseline filtering, peak identification, integration, retention time correction, peak alignment, and other processing steps, ultimately yielding a matrix containing information such as retention time, mass‐to‐charge ratio, and peak intensity. Subsequently, the software was used to identify characteristic peaks by searching the metabolite database and matching MS and MS/MS data with the metabolite database. The MS mass error was set to less than 10 ppm, and metabolites were identified on the basis of the MS/MS matching score. Principal component analysis (PCA) and orthogonal least squares discriminant analysis (OPLS‐DA) were performed on the preprocessed data matrix, and the model stability was evaluated using 7 rounds of cross‐validation. Significantly differentially expressed metabolites were identified on the basis of the variable importance (VIP) values obtained from the OPLS‐DA model and the *p* values from Student's *t*‐tests. When the VIP was > 1 and *p* was < 0.05, the metabolite was considered to be differentially expressed.

### 2.7 Ethics

This study was approved by an independent ethics committee (Ethics Committee of Yunnan Cancer Hospital), who reviewed the procedures for the collection, storage, and use of the data and samples as well as related documents to ensure that the rights, safety, and health of the participants were properly protected. Before the study began, the researchers clearly explained the purpose, content, and significance of the study to the participants and obtained their written informed consent. During the intervention, the researchers adhered to the principle of voluntary participation by the individuals, fully respected their individual wishes, and protected their personal information.

### 2.8 Statistical Analysis

The data were analyzed primarily with SPSS 26.0 and GraphPad Prism 8.0. The Shapiro–Wilk test was used for normality testing, and the Levene test was used to evaluate the homogeneity of variables. The Mann–Whitney rank‐sum test was used to analyze skewed data. Fisher's exact test was used for count data. Pearson's correlation coefficient was calculated to assess correlations for normally distributed variables, and Spearman correlation coefficient was used to assess correlations for nonnormally distributed data. *p* < 0.05 was considered to indicate statistical significance.

## 3 Results

### 3.1 Baseline Data

**FIGURE 1:**
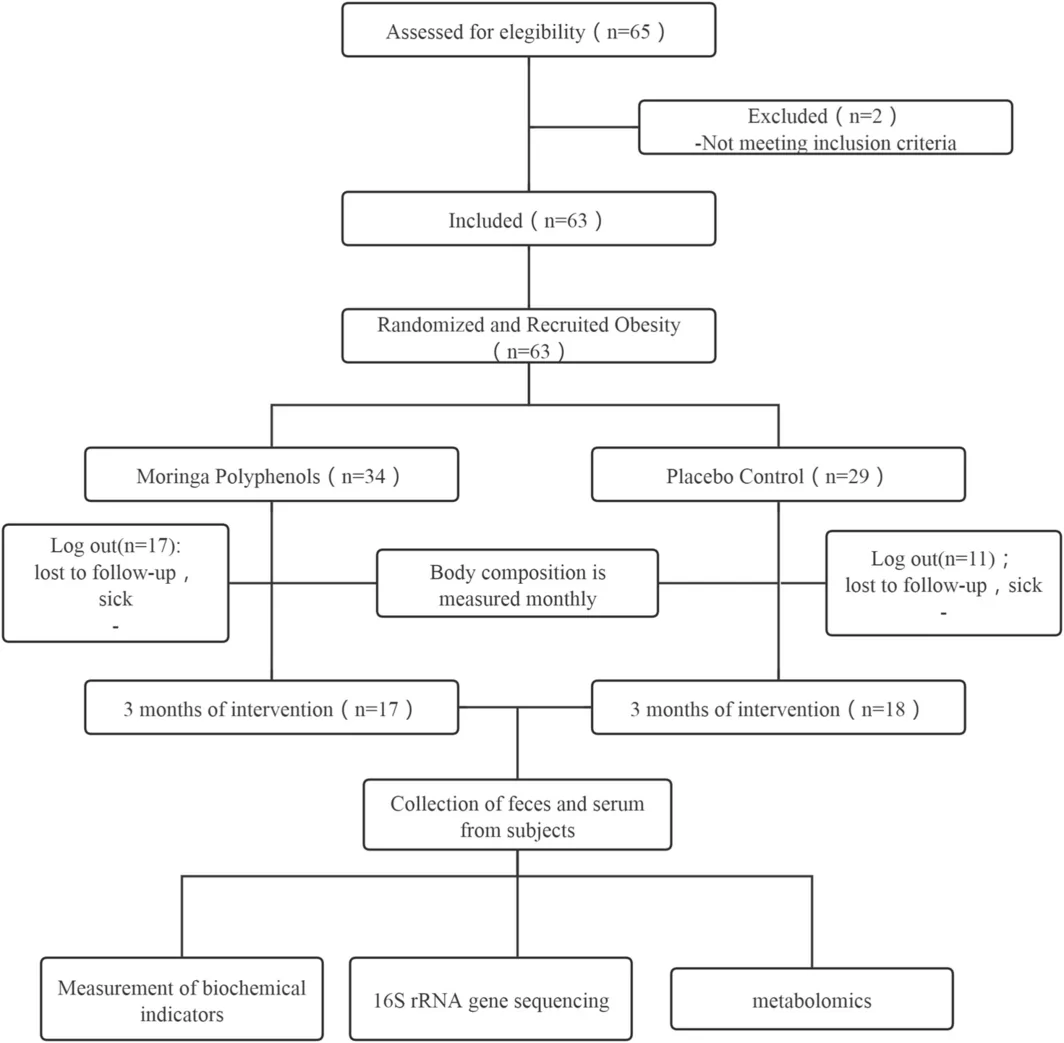
Flowchart of the study procedures. The body composition of each patient was measured once every month. Some patients missed the first or second body composition measurements for various reasons, such as business trips, being busy with work, long distances, and illness. Therefore, during the first month, we collected data from 19 participants in the Con group and 24 in the Med group. In the second month, we obtained data from 14 participants in the Con group and 19 in the Med group.

The protocol of this study is illustrated in Figure 1. Of the 65 volunteers initially recruited, two were deemed ineligible and thus excluded from the study. The 63 remaining volunteers were randomly assigned to the Con or Med group. The baseline information of the volunteers is shown in Table 3; these values include only information from the 35 volunteers for whom complete data are available. In the Con and Med groups, the median ages were 35 and 38.32 years, respectively; the ratios of females to males were 11/18 and 13/21, respectively; and the BMIs of all participants were greater than or equal to 28, with medians of 29.4 and 30.5, respectively. There were no significant differences in the baseline characteristics between the groups.

**TABLE 3:** Baseline information of the participants in the Med and Con groups.

Variable	Control (*n* = 29)	Intervention (*n* = 34)	*p*
Demographic information			
Age (y)	35 (26.5, 39.5)	38.32 ± 9.26	0.11
Sex (female/male)	18/11	21/13	1.00
Liver function variables			
Bile acid (μmol/L)	2.2 (1.3, 3.9)	2.7 (1.38, 4.6)	0.28
Alanine transaminase (U/L)	19.5 (14, 23.25)	17.85 (14.3, 23.63)	0.79
Glutamine aminotransferase (U/L)	23.6 (14.5, 36.75)	23.85 (16.18, 36.85)	0.99
Total bilirubin (μmol/L)	10.08 ± 4.06	8.35 (6.8, 10.45)	0.42
Direct bilirubin (μmol/L)	3.4 (2.9, 4.65)	3.2 (2.8, 4.18)	0.29
Indirect bilirubin (μmol/L)	6 (3.9, 7.9)	5.2 (4.15, 6.48)	0.48
Total protein (g/L)	75.81 ± 3.58	74.93 ± 2.47	0.26
Albumin (g/L)	47.90 ± 2.68	47.45 (46.22, 49.7)	0.58
Globulin (g/L)	27.91 ± 3.15	26.85 (24.86, 28.66)	0.23
Prealbumin (mg/L)	276.98 ± 39.64	278.9 (239.15, 305.475)	0.63
Blood lipid variables			
Triglycerides (mmol/L)	1.87 (1.43, 2.46)	1.68 (1.24, 2.58)	0.66
Total cholesterol (mmol/L)	5.01 ± 0.81	4.70 (4.4, 5.23)	0.17
Low‐density lipoprotein (mmol/L)	3.09 ± 0.75	2.95 ± 0.57	0.41
Apolipoprotein‐A1 (g/L)	1.37 ± 0.20	1.34 ± 0.17	0.51
Apolipoprotein‐B (g/L)	1.07 ± 0.21	1.03 ± 0.18	0.35
CRP			
C‐reactive protein (mg/L)	1.74 (1, 3.01)	2.59 (1.07, 5.44)	0.28
Kidney function parameters			
Urea (μmol/L)	4.63 ± 1.07	4.83 (4.34, 5.60)	0.13
Creatinine (μmol/L)	74.43 ± 12.37	73.03 ± 13.18	0.67
Uric acid (μmol/L)	426.25 ± 85.47	401.73 ± 70.30	0.24
Glycemic parameters			
Glycosylated hemoglobin (%)	5.74 ± 0.33	5.93 ± 0.42	0.055
Glucose (mmol/L)	4.92 (4.69, 5.1)	5.08 ± 0.60	0.48
Body composition			
Weight (kg)	85 (73.25, 91.5)	85.5 (69.98, 96.75)	0.60
Height (cm)	165.67 ± 8.50	165.82 ± 7.93	0.94
BMI	29.4 (28.55, 33)	30.5 (28.65, 33.33)	0.41
Waist circumference (cm)	101 (93.75, 104.25)	100 (95.75,115)	0.60
Hip circumference (cm)	108 (103, 113)	106 (103, 112)	0.53
Waist‐to‐hip ratio	92.33 ± 6.79	96.34 ± 7.67	0.94
Fat percentage (%)	39.08 ± 9.41	41.1 ± 7.30	0.43
Visceral fat grade	12.82 ± 3.72	13 (9, 20.25)	0.28
Basal metabolic load (kcal)	1559.27 ± 216.46	1582.11 ± 259.39	0.74
Other variables			
Homocysteine (μmol/L)	13.33 ± 2.84	12.7 (9.8, 15.25)	0.38
Leukocyte count (10^9^/L)	7.56 (6.12, 8.47)	7.51 ± 2.40	0.52
Absolute lymphocyte count (10^9^/L)	2.59 ± 0.81	2.57 ± 0.72	0.93
Neutrophil count (10^9^/L)	4.05 ± 1.57	4.02 (2.73, 5.03)	0.98
Absolute erythrocyte count (10^9^/L)	5.11 ± 0.40	5.00 ± 0.40	0.28
Platelet count (10^9^/L)	305 (224.5, 336)	281.82 ± 76.57	0.42

*Note:* Count data are presented as frequencies, normally distributed data are presented as ($\bar{x}$ ± *s*), and skewed data are presented as *P*_*50*_ (*P*_*25*_, *P*_*75*_). *p* < 0.05 was considered to indicate statistical significance difference between the groups.

### 3.2 Body Composition

**FIGURE 2:**
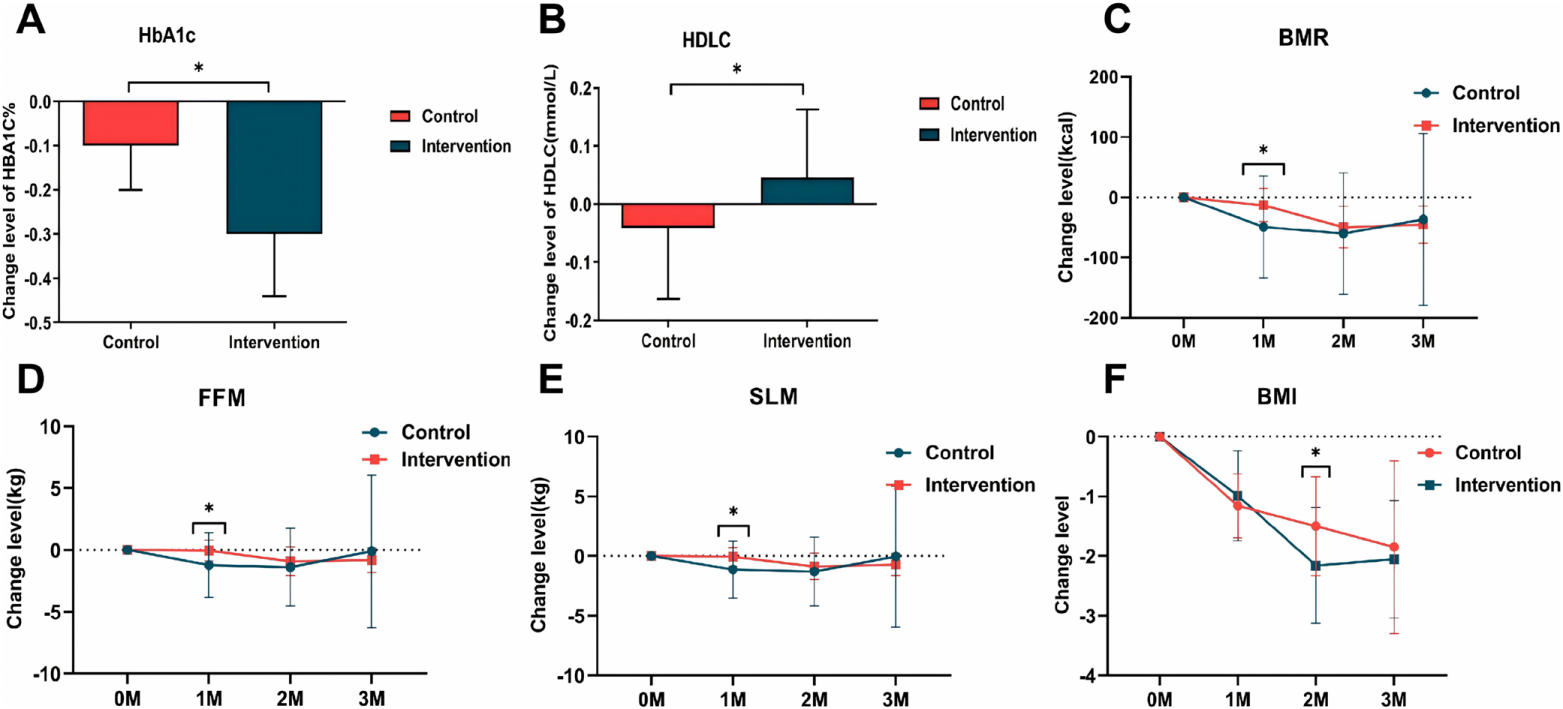
Changes in outcome indicators. (A) Glycosylated hemoglobin (HbA1c). (B) High‐density lipoprotein (HDL‐C). (C) Basal metabolic rate (BMR). (D) Fat‐free weight (FFM). (E) Muscle mass (SLM). (F) BMI. *Indicates *p* < 0.05. In figures (A) and (B), control *n* = 17, intervention *n* = 17. In the figures (C–F), control *n* = 19 and intervention *n* = 24 at 1 M; control *n* = 14 and intervention *n* = 19 at 2 M; control *n* = 18 and intervention *n* = 15 at 3 M.

During the trial, each participant's body composition data were measured once a month. After 1 month, compared with those at baseline, fat‐free body weight, muscle mass, and basal metabolic rate decreased by 0.65 kg, 0.6 kg and 49.1 kcal, respectively, in the Con group and by 0.06 kg, 0.06 kg, and 13.12 kcal, respectively, in the Med group; additionally, fat‐free body weight, muscle mass, and basal metabolic rate were significantly lower in the Med group than in the Con group (Figure 2C–E; *p* < 0.05). After 2 months, the BMI decreased by 1.5 kg/m^2^ and 2.16 kg/m^2^ in the Con and Med groups, respectively, relative to those at baseline; the decrease in BMI was significantly greater in the Med group than in the Con group (Figure 2F, *p* < 0.05). After 3 months, in the Con and Med groups, the fat percentage decreased by 2.75 percentage points and 3.22 percentage points, the waist‐to‐hip ratio decreased by 0.78 and 2.25, the visceral fat grade decreased by 1.44 and 2, and the basal metabolic score increased by 0.9 and 2, respectively. These results suggest that weight loss, a lower fat percentage, waist‐to‐hip ratio and visceral fat grade, and a higher basal metabolic rate were more common in the Med group than in the Con group.

### 3.3 Lipids

After 3 months, there was a significant difference in the average change in the serum HDL‐C levels of the participants in the two groups. Specifically, the HDL‐C level decreased by 0.04 mmol/L in the Con group but increased by 0.05 mmol/L in the Med group (Figure 2B, *p* < 0.05). No significant differences were found among the other lipid indices (*p* > 0.05).

### 3.4 Blood Sugar

At the end of the 3‐month intervention, there was a significant difference in the average HbA1c level between the two groups (Figure 2A, *p* < 0.05). Compared with that at baseline, the HbA1c level at 3 months decreased by 0.1% and 0.31% in the Con group and Med group, respectively. These findings indicate that *M. oleifera* leaf extract may decrease HbA1c levels. Compared with that at baseline, the fasting blood glucose level of the patients in the Con group increased by 0.07 mmol/L but decreased by 0.26 mmol/L in the Med group, although the difference was not significant (*p* > 0.05).

### 3.5 16S rRNA Gene Sequencing

**FIGURE 3:**
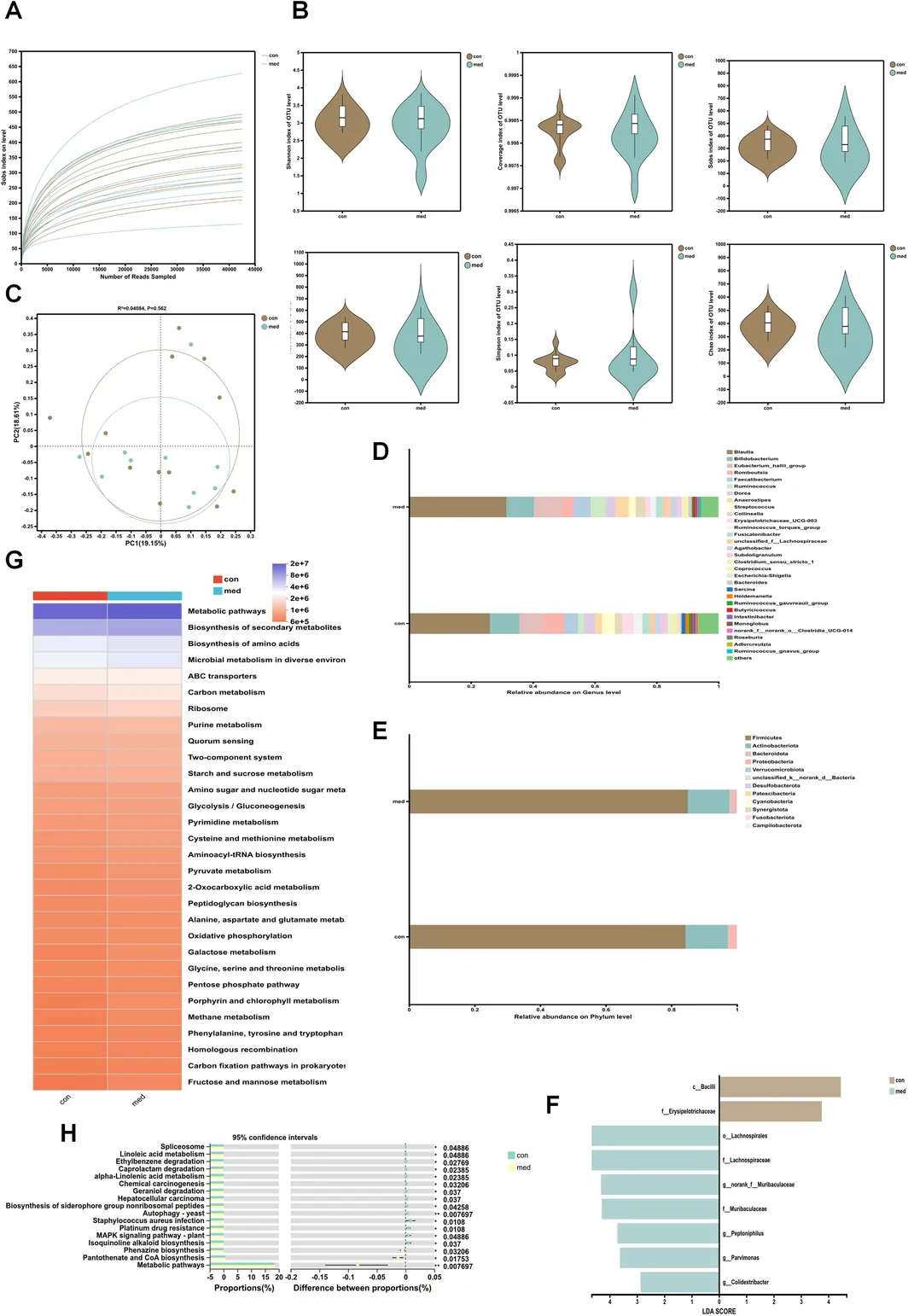
Microbial diversity. (A) Dilution curve. (B) Diversity index. (C) PCoA of the taxonomic OTU level. (D) Histogram of microbial composition at the genus level. (E) Histogram of microbial composition at the phylum level. (F) LDA bar chart. (G) PICRUSt2 functional prediction for KEGG pathways. (H) Significantly differentially enriched KEGG pathways between the groups. *Control *n* = 13, Intervention *n* = 11.

A total of 1,503,258 raw sequences were obtained from the 24 samples at a sequencing length of 300 paired‐end reads. We obtained 1,414,458 optimized sequences, the average length of which was 408. The dilution curves indicated sufficient sequencing depth (Figure 3A). The *p* values for six α‐diversity indices were greater than 0.05, indicating that there were no significant differences in species diversity, community richness, or evenness between the two groups (Figure **3**B). In the PCoA plot depicting β‐diversity (Figure 3C), the two groups tended to overlap, and no clear separation was evident. The *p* value (0.517) was greater than 0.05, indicating that there was no significant difference in the overall community structure between the two groups. At the phylum level, the main flora in both groups were *Firmicutes*, *Actinobacteria*, *Proteobacteria*, and *Bacteroidetes* (Figure 3E). At the genus level, the dominant flora were *Blautia*, *Bifidobacterium*, *Eubacterium_hallii_group*, *Romboutsia*, and others (Figure 3D). Beneficial bacteria, such as Christensenellaceae, Lactobacillus, and Bifidobacterium, were less abundant in the Med group than in the Con group, whereas Porphyromonadaceae and Rikenellaceae were more abundant in the Con group. Although there were no notable differences in species abundance between the two groups, there were marked differences in the abundances of nine taxa (one order, one phylum, three families, and four genera) (Figure 3F). Among these taxa, Bacillus (*p* < 0.05, LDA = 4.45) and Erysipelotrichaceae (*p* < 0.05, LDA = 3.75) were more abundant in the Con group, and Lachnospirales (*p* < 0.05, LDA = 4.66), Lachnospiraceae (*p* < 0.05, LDA = 4.66), norank_f__Muribaculaceae (*p* < 0.05, LDA = 4.32), Muribaculaceae (*p* < 0.05, LDA = 4.29), Peptoniphilus (*p* < 0.05, LDA = 3.72), Parvimonas (*p* < 0.05, LDA = 3.63), and Colidextribacter (*p* < 0.01, LDA = 2.87) were more abundant in the Med group. According to PICRUSt2 functional predictions, the functional abundances of the KEGG Level 3 pathways “Metabolic pathways,” “Pantothenate and CoA biosynthesis,” and “Phenazine biosynthesis” were significantly greater in the Med group than in the Con group, whereas the functional abundances of pathways including “Linoleic acid metabolism,” “Alpha‐linolenic acid metabolism,” “Chemical carcinogenesis,” and “Hepatocellular carcinoma,” were significantly lower in the Med group than in the Con group.

### 3.6 Short‐Chain Fatty Acids (SCFAs)

**FIGURE 4:**
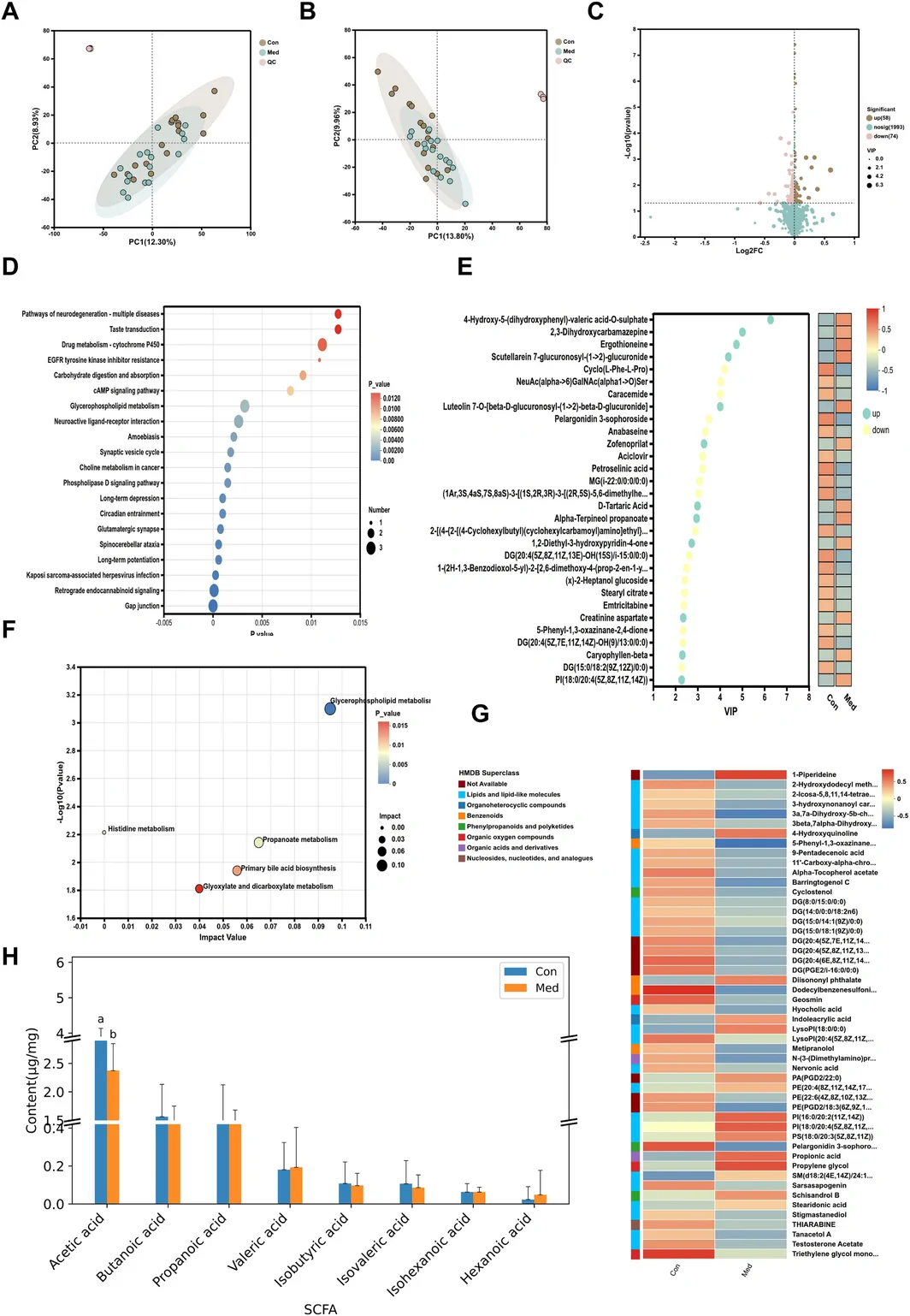
Untargeted metabolomic and SCFA analysis. (A) Positive ion PCA score plot. (B) Negative ion PCA score plot. (C) Volcano map of the differentially abundant metabolites. (D) KEGG pathway enrichment bubble map. (E) Metabolites with the top 30 VIP scores. (F) KEGG topological analysis of the bubble diagram. (G) Cluster analysis of the top 50 differentially abundant metabolites by HMDB superclass. (H) Histogram of short‐chain fatty acid abundance. *In panels (A–G), control *n* = 17, intervention *n* = 16; in panel (H), control *n* = 13, intervention *n* = 11.

The levels of SCFAs were lower in the Med group than in the Con group (Figure 4H). The levels of acetic acid were significantly lower in the Med group than in the Con group (*p* < 0.05), but the contents of other SCFAs, such as propionic, butyric, isobutyric, isovaleric, valeric, isohexanoic, and caproic acids, did not differ between the groups.

### 3.7 Untargeted Metabolomics Analysis

As shown in Figure 4A,B, the points for both the control group and the *M. oleifera* leaf extract group in the cation–anion performance chart fall within the 95% confidence interval ellipse, indicating the absence of outliers and good biological reproducibility. However, the differences between the two groups are not clear. Databases such as Metlin and HMDB were used to analyze the 903 and 1222 metabolites identified in positive and negative ion modes, respectively; the results revealed that 54 and 78 metabolites in positive and negative ion modes, respectively, were differentially abundant between the two groups (Figure 4C). These metabolites were significantly enriched in five metabolic pathways: glycerophospholipid (GP) metabolism, propionic acid metabolism, primary bile acid biosynthesis, glyoxylate and dicarboxylic acid metabolism, and histidine metabolism (Figure 4F). According to Figure 4E, metabolites with VIP values greater than 4 included 4‐hydroxy‐5‐(dihydroxyphenyl)‐valeric acid‐O‐sulfate, 2,3‐dihydroxycarbamazepine, ergothioneine, scutellarein 7‐glucuronosyl‐(1‐> 2)‐glucuronide, cyclo(L‐Phe‐L‐Pro), NeuAc(α‐> 6)GalNAc(α1‐>O)Ser, caracemide, and luteolin 7‐O‐[β‐D‐glucuronosyl‐(1‐> 2)‐ β‐D‐glucuronide]. These eight metabolites may play major roles in the effects of *M. oleifera* leaf extract. Cluster analysis (shown in Figure 4G) shows the changes in the top 50 metabolites ranked by differential abundance between the two groups. In terms of lipids and lipid‐like molecules, compared with those in the Con group, the levels of diacylglycerol (DG), 3a,7a‐dihydroxy‐5b‐cholestane, 3‐hydroxynonanoyl carnitine, and 2‐Icosa‐5,8,11,14‐tetraen‐2‐yloxypropane‐1,3‐diol were significantly lower in the Med group. In contrast, the levels of phosphatidylinositol (PI), propionic acid, propylene glycol, and SM(d18:2(4E,14Z)/24:1(15Z)) were significantly greater in the Med group.

### 3.8 Correlation

**FIGURE 5:**
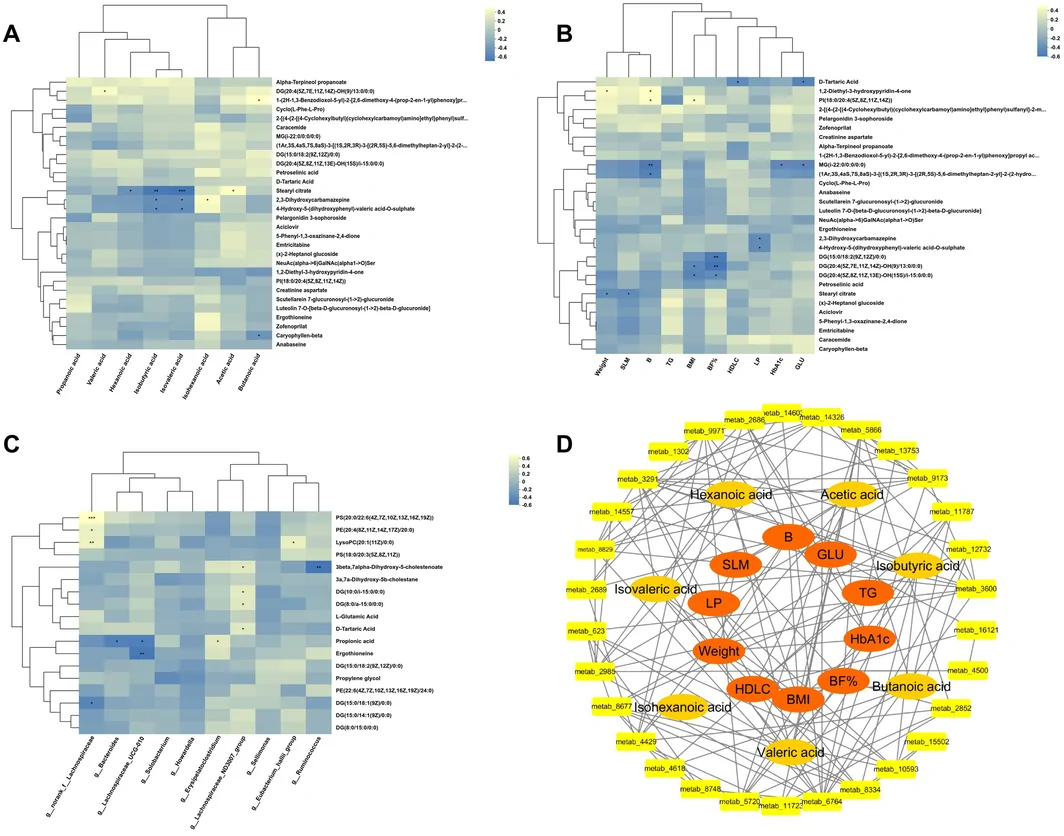
Correlation analysis of omics data and clinical data. (A) Heatmap of the correlation between SCAF and the top 30 metabolites in VIP. (B) Heatmap showing the correlation between selected clinical data and the top 30 differential metabolites in the VIP cohort. (C) Heatmap showing the correlation between the horizontal microbial community and the top 30 VIP metabolites. (D) Correlation network diagram.

As shown in Figure 5, some correlations among the enriched metabolites and clinical biochemical indicators were noted. For example, the level of HDL‐C was negatively correlated with those of DG (10:0/i‐15:0/0:0) (*r* = −0.3952, *p* < 0.05) and DG (8:0/a‐15:0/0:0) (*r* = −0.4623, *p* < 0.01); the level of TG was positively correlated with that of DG (8:0/a‐15:0/0:0) (*r* = 0.3577, *p* < 0.05); and the level of HbA1c was positively correlated with the levels of 3α,7α‐dihydroxy‐5β‐cholestane (*r* = 0.3644, *p* < 0.05) and DG (8:0/a‐15:0/0:0) (*r* = 0.3832, *p* < 0.05). There were also some correlations among the top 30 differentially abundant metabolites (on the basis of the VIP value) and clinical indicators. For example, the BMI was positively correlated with the levels of HbA1c (*r* = 0.4531, *p* < 0.05), PI (18:0/20:4(5Z,8Z,11Z,14Z)) (*r* = 0.4185, *p* < 0.05), and DG (20:4(5Z,7E,11Z,14Z)‐OH(9)/13:0/0:0) (*r* = −0.4428, *p* < 0.05) and negatively correlated with the level of DG (20:4(5Z,8Z,11Z,13E)‐OH(15S)/i‐15:0/0:0) (*r* = −0.4733, *p* < 0.05) (Figure 5B,C); muscle mass was negatively correlated with the levels of leptin (*r* = −0.4479, *p* < 0.05), acetic acid (*r* = −0.4634, *p* < 0.05), and stearyl citrate (*r* = −0.4235, *p* < 0.05); the level of HDL‐C was negatively correlated with the level of acetic acid (*r* = −0.4526, *p <* 0.05), butyric acid (*r* = −0.4182, *p* < 0.05) and D‐tartaric acid (*r* = −0.4321, *p* < 0.05); the level of HbA1c was negatively correlated with the level of MG (i‐22:0/0/0:0/0:0) (*r* = −0.4468, *p* < 0.05); and the level of acetic acid was positively correlated with the level of stearyl citrate (*r* = 0.4339, *p* < 0.05). A correlation analysis was conducted on key metabolites within gut microbial communities and metabolic pathways that have been extensively studied in relation to disease. The analysis revealed a significant correlation between them, such as between *Lachnospiraceae* and PS (20:0/22:6(4Z,7Z,10Z,13Z,16Z,19Z)) (*r* = 0.6381, *p* < 0.05), PE (20:4(8Z,11Z,14Z,17Z)/20:0) (*r* = 0.4966, *p* < 0.05), LysoPC (20:1(11Z)/0:0) (*r* = 0.521, *p* < 0.01), DG (15:0/18:1(9Z)/0:0) (*r* = −0.4183, *p* < 0.05) showed a negative correlation; *Bacteroides* showed a negative correlation with propionic acid (*r* = −0.4579, *p* < 0.05); *Lachnospiraceae_UCG‐010* showed a negative correlation with propionic acid (*r* = −0.4675, *p* < 0.05) and ergothioneine (*r* = −0.5311, *p* < 0.01); *Erysipelatoclostridium* showed a positive correlation with propionic acid (*r* = 0.4409, *p* < 0.05); *Lachnospiraceae_ND3007_group* was negatively correlated with 3β,7α‐dihydroxy‐5‐cholestenoate (*r* = 0.4274, *p* < 0.05), DG (10:0/i‐15:0/0:0) (*r* = 0.4515, *p* < 0.05), DG (8:0/a‐15:0/0:0) (*r* = 0.4225, *p* < 0.05), and D‐tartaric acid (*r* = 0.4153, *p* < 0.05).

## 4 Discussion

In China, *M. oleifera* leaves have recently emerged as a novel food resource that exhibits significant antiglycaemic and anticancer properties, as evidenced in prior studies (Cava et al. 2017; Mthiyane et al. 2022). In recent years, many studies have focused on the antiobesity effects of these leaves in animal and in vitro experiments, but few investigations have assessed these effects in humans. In vitro and in vivo studies have shown that *M. oleifera* leaf extract may be promising for preventing and controlling obesity (Zhang et al. 2021; Cho et al. 2025).

This study was a randomized, double‐blind, placebo‐controlled clinical trial, the results of which indicated that participants in both the Con and Med groups experienced significant weight loss, but the weight loss was more pronounced in the Med group, especially during the second month of the intervention. We found that the participants who rapidly lost weight adhered less to the treatment regimen and tended to take the *M. oleifera* leaf extract intermittently, which may have led to the lack of significance in the difference in weight loss between the two groups by the third month. Additionally, participants in the Med group demonstrated superior fat‐free body weight retention at the end of the first month, but this advantage was not sustained over the subsequent 2 months; thus, further studies are needed to fully assess these effects.

*M. oleifera* leaf extract has been shown to have hypoglycaemic effects in various animal models and clinical studies involving T2DM patients (Zaplana et al. 2023; Gómez‐Martínez et al. 2021; Panova et al. 2025), as the extract effectively decreased participants' fasting blood glucose and HbA1c levels. At the same time, *M. oleifera* leaves can also improve blood lipid profiles, such as HDL‐C (Liu et al. 2026). In our trial, as the HDL‐C level increased, the HbA1c level of the participants significantly decreased in the Med group. Our results showed that *M. oleifera* leaf extract could influence HbA1c and HDL‐C levels in individuals with simple obesity, which may indicate that it could not only aid in body weight reduction but also improve blood glucose and lipid profiles. We believe that *M. oleifera* leaves may be a promising substance for preventing and controlling simple obesity, diabetes, and dyslipidemia.

The gut microbiota plays a pivotal role in the development of obesity by modulating energy absorption, lipid metabolism, appetite, and inflammatory responses. In our study, we observed that *M. oleifera* leaf extract notably modified the composition of the gut microbiota in individuals with simple obesity; in the Med group, the abundances of *Lachnospiraceae* and *Muribaculaceae* were elevated, while that of *Erysipelotrichaceae* was diminished. In a study conducted by Chitra Nehra et al. on the growth‐promoting effects of moringa leaves in ruminants, significant changes were observed in the *Lachnospiraceae* of the *M. oleifera* group (Nehra et al. 2024). *Muribaculaceae* and *Lachnospiraceae* are considered beneficial bacteria. The former is thought to play a role in mitigating inflammatory responses and inhibiting harmful bacteria. Some studies have shown that *M. oleifera* leaf extract may exert anti‐inflammatory effects by increasing the abundance of *Lachnospiraceae* (Hong et al. 2022). *Lachnospiraceae* are integral to the intestinal health of individuals and help mitigate the risk of obesity (You et al. 2022; Li et al. 2024). Furthermore, *Lachnospiraceae* are also principal producers of SCFAs, primarily butyrate and propionate (Vacca et al. 2020; Martin‐Gallausiaux et al. 2021). SCFAs, including acetic acid, propionic acid, and butyric acid, are the primary end products of dietary fiber fermentation in the gut. Recent studies have demonstrated that SCFAs can fortify the intestinal barrier, promote beneficial bacterial proliferation, and bolster the immune system (Koh et al. 2016; Grosshagauer et al. 2021); however, other studies have shown opposite results. Given the close association between *Lachnospiraceae* and SCFAs, we hypothesize that *M. oleifera* leaf extract alters metabolism in consumers by regulating SCFAs. Compared with that in the Con group, the content of acetic acid in the feces of the participants in the Med group was lower, whereas the contents of the other SCFAs were not significantly altered. These findings are consistent with the changes in SCFA levels observed in patients who lose weight with change their diets (Sowah et al. 2020; Meijer et al. 2022). Therefore, the *M. oleifera* leaf extracts may be associated with the changes in SCFAs observed in the participants in the Med group, but further research is needed to verify this conclusion. Acetic acid has a bidirectional effect on the human body: it can not only suppress appetite and help weight loss but also act as a substrate for cholesterol synthesis and promote obesity (Geng et al. 2022). Clinical studies have shown that levels of fecal acetate and total SCFAs are significantly higher in obese individuals and people with diabetes than in healthy individuals (Dede et al. 2025). Some studies have shown that a decrease in SCFA levels may predict poor weight loss outcomes, but in the present study, the level of acetic acid was moderately negatively correlated with muscle mass and the level of HDL‐C. In our study, the level of HDL‐C increased significantly, and muscle mass was better retained when the level of acetic acid was reduced in the patients in the Med group. Therefore, the decrease in acetic acid level caused by consumption of the *M. oleifera* leaf extract may be beneficial.

The metabolomics results revealed enrichment of the differentially abundant metabolites in five main metabolic pathways: GP metabolism, propionic acid metabolism, primary bile acid biosynthesis, glyoxylate and dicarboxylic acid metabolism, and histidine metabolism. GPs are intricately linked to metabolism; some studies have indicated that lysophosphatidylcholine (LPC) may be negatively correlated with obesity, atherosclerosis, insulin resistance, and inflammation. Additionally, lysophosphatidylethanolamine (LPE), phosphatidylcholine prophosphatidylcholines (PC‐PLs), and sphingomyelins (SMs) are thought to be negatively associated with the risk of T2DM; however, diacylglycerols (DGs) and phosphatidylethanolamine (PE) are positively correlated with the risk of T2DM. In this study, the levels of partial glycerophosphate (PS), phosphatidylcholine (PC), and LPC tended to increase, suggesting potential associations with weight loss.

DGs are pivotal molecules in numerous metabolic pathways (Luo et al. 2022; Fang et al. 2022; Surowiec et al. 2019). In our research, compared with those in the Con group, the levels of DGs comprehensively decreased in the Med group. Correlation analysis revealed that the level of HDL‐C was negatively correlated with that of DG (10:0/i‐15:0/0/0:0) and DG (8:0/a‐15:0/0:0). Conversely, the levels of HbA1c and serum TG were positively correlated with the level of DG (8:0/a‐15:0/0:0). These findings suggest that the changes in HDL‐C and HbA1c levels in the Med group may be associated with a decrease in the levels of DGs. Furthermore, histidine metabolism is strongly associated with obesity (Singh et al. 2023). The level of acylcarnitine is positively correlated with the risk of metabolic disorders such as obesity and insulin resistance. In this study, the levels of heptadecanoyl carnitine and 3‐hydroxynonoyl carnitine tended to be lower in the Med group, indicating their potential association with changes in BMI, HDL‐C, and HbA1c. Additionally, after the participants consumed *M. oleifera* leaf extract, among the metabolites whose VIP values significantly differed, MG (i‐22:0/0:0/0:0) was negatively associated with HbA1c. Furthermore, the level of stearyl citrate was negatively correlated with muscle mass and positively correlated with the level of acetic acid, whereas the level of D‐tartaric acid was negatively correlated with that of HDL‐C. These results suggest that these metabolites may also be associated with the effects of *M. oleifera* leaf extract on HbA1c, muscle mass, and HDL‐C. However, further research is needed.

## 5 Conclusion

In our study, *M. oleifera* leaf extract effectively decreased BMI while maintaining basal metabolic rate, muscle mass, and fat‐free mass, and simultaneously lowered HbA1c and increased HDL‐C levels in individuals with simple obesity. In addition, the extract increased the relative abundance of *Lachnospiraceae* and *Muribaculaceae*, decreased the relative abundance of *Erysipelotrichaceae*, and significantly reduced the level of fecal acetic acid. Glycolipid metabolic pathways were enriched in the differentially abundant metabolites. Some correlations were identified among the aforementioned indicators, which may represent that *M. oleifera* leaf extract has a certain regulatory effect on the gut microbiota and glucose and lipid metabolism in obese individuals.

## Author Contributions

**Daoqiang He:** resources, data curation. **Xiaoxian Zhou:** validation, formal analysis, investigation, writing – original draft, visualization. **Huilin Chen:** data curation. **Jia Xiong:** validation, resources. **Lan Zhou:** data curation. **Xinle Zhang:** writing – review and editing. **Jia Luo:** validation, resources. **Fei Mi:** methodology. **Shaoxiong Wu:** conceptualization, supervision, funding acquisition. **Hongmei Pan:** conceptualization, writing – review and editing, supervision. **Yanjiao Wang:** methodology, project administration. **Jingwen Dao:** data curation. **Yongfang Zhou:** software.

## Funding

This work was supported by the Key Special Project Program of Yunnan Provincial Department of Science and Technology (202102AE090027).

## Ethics Statement

The study was conducted in accordance with the Declaration of Helsinki and approved by the Ethics Committee of Yunnan Cancer Hospital (protocol code: KYLX2022179; date: 14 October 2022) and the Ethics Committee of Kunming Medical University (protocol code: KMMU2020MEC074; date: 04 August 2020). All participants provided written informed consent. All volunteers provided written informed consent to participate in the study.

## Consent

The authors have nothing to report.

## Conflicts of Interest

The authors declare no conflicts of interest.

## Supporting information


**Table S1:** fsn372292‐sup‐0001‐TableS1.doc.

## Data Availability

The data that support the findings of this study are available in NCBI SRA database (PRJNA1462614) and OMIX database of the National Center for Bioinformation (https://ngdc.cncb.ac.cn/omix/preview/7cr7s9HJ, OMIX016846).
