# Supplementary material for: Impacts of M. oleifera Leaf Extract on Weight Loss, the Metabolomic Profile, and the Intestinal Microbiota in Individuals With Simple Obesity
Source: Food Sci Nutr. 2026 Aug 28;14(9):e72292. doi: 10.1002/fsn3.72292 (PMC13522931; doi:10.1002/fsn3.72292)
Supplement: Supplementary file 1 [file FSN3-14-e72292-s001.doc]

**Table S1**: Laboratory Methods

| **Indicators** | **Methods** |
| --- | --- |
| Bile acid (μmol/L) | Cyclic Enzyme Method |
| Alanine transaminase (U/L) | Rate Method |
| Glutamine aminotransferase (U/L) | Rate Method |
| Total bilirubin (μmol/L) | Diazo Method |
| Direct bilirubin (μmol/L) | Diazo Method |
| Indirect bilirubin (μmol/L) | Calculation Method |
| Total protein (g/L) | Biuret Method |
| Albumin (g/L) | Bromocresol Green Method |
| Globulin (g/L) | Calculation Method |
| Prealbumin (mg/L) | Immunoturbidimetric Assay |
| Triglycerides (mmol/L) | Enzymatic Method |
| Total cholesterol (mmol/L) | Oxidase Method |
| Low-density lipoprotein (mmol/L) | Direct Method |
| Apolipoprotein-A1 (g/L) | Immunoturbidimetric Assay |
| Apolipoprotein-B (g/L) | Immunoturbidimetric Assay |
| C-reactive protein (mg/L) | Immunoturbidimetric Assay |
| Urea (μmol/L) | Urease Colorimetric Assay |
| Creatinine (μmol/L) | Enzymatic Method |
| Uric acid (μmol/L) | Colorimetric Uricase Assay |
| Glycosylated haemoglobin (%) | High-Performance Liquid Chromatography |
| Glucose (mmol/L) | Hexokinase Assay |
| Homocysteine (μmol/L) | Enzymatic Method |
